# Occult lung malignancy presenting with finger pain: a case report

**DOI:** 10.1186/1752-1947-2-364

**Published:** 2008-12-04

**Authors:** Matthew A Embley, Rebecca B Goody, Mahmood Mughrabi

**Affiliations:** 1 Department of Respiratory Medicine, University of Glasgow, Ward 6C, Gartnavel General Hospital, Great Western Road, Glasgow, G12 0YN, UK; 2 Respiratory Unit, Victoria Hospital, Hayfield Road, Kirkcaldy, Fife, KY2 5AH, UK

## Abstract

**Introduction:**

Lung cancer is currently one of the most common malignancies in the world. Early detection is an important prognostic factor. Unfortunately, initial symptoms may be vague and a substantial proportion of cases present with the effects of metastases.

**Case presentation:**

We discuss a case of occult lung malignancy in a 61-year-old man. The only symptom at presentation was pain in the right ring finger due to metastasis from the lung primary.

**Conclusion:**

This case highlights the need for vigilance when a patient presents with unusual or unexplained symptoms, especially if they have known risk factors for cancer.

## Introduction

The American Cancer Society estimates that lung cancer remains the most common malignancy in men and fourth most common in women worldwide. These global estimates also place lung malignancy as the most common cause of cancer death in men and second most common in women [1]. Despite its prevalence, little progress has been made over the past 30 years to improve prognosis. Five-year survival rates are currently around 10% [2,3]. Cigarette smoking is the most well known aetiological factor, with an estimated 10% of smokers developing the disease [2]. One of the most important factors in determining prognosis is the stage of disease with which patients present [2,3]. Stage III and IV disease account for more than three-quarters of new diagnoses; the late presentation is often due to lack of physical symptoms in earlier stages of disease or patient delay in seeking medical review [4].

Lung cancer can present with respiratory symptoms including dyspnoea, cough and haemoptysis. Other symptoms at presentation include hoarseness, weight loss, and chest and bone pain [4]. Asymptomatic lesions may be detected on radiological investigations performed for other reasons. An estimated 60% of patients with small cell, and 40% with non-small cell lung cancer present with distant metastases [5].

## Case presentation

**Figure 1:**
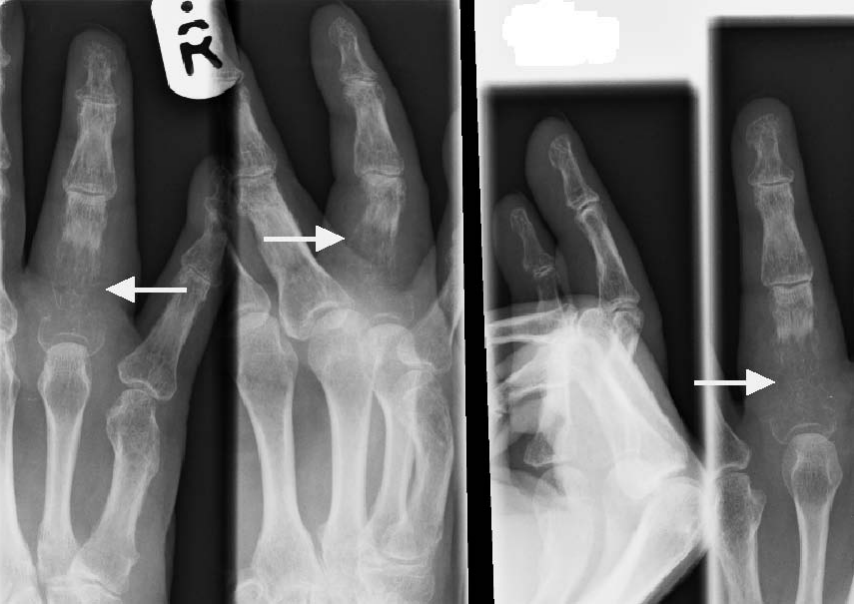
X-Ray of the right hand showing destruction of the proximal phalanx of the ring finger (arrows).

A 61-year-old man presented to his general practitioner with a short history of pain in his right ring finger. His finger was swollen and tender particularly in the proximal phalanx. Treatment was initially with non-steroidal anti-inflammatory drugs and investigation was performed for possible gout. As his pain failed to improve in the following month, he was referred for an X-ray of his right hand (Figure 1).

This was reported as showing significant translucency of the proximal phalanx of the right ring finger. Features were felt to be consistent with an aggressive infective process, although the radiologist could not exclude non-benign infiltration. The patient was initially referred for an orthopaedic opinion. At the time of review, he denied any weight loss or other bony symptoms; systemic enquiry was unremarkable and of particular note he had no respiratory symptoms, other than a longstanding non-productive cough. He was a heavy smoker of up to 40 cigarettes per day, with moderate alcohol consumption and a past medical history of cerebrovascular disease and hypertension.

**Figure 2:**
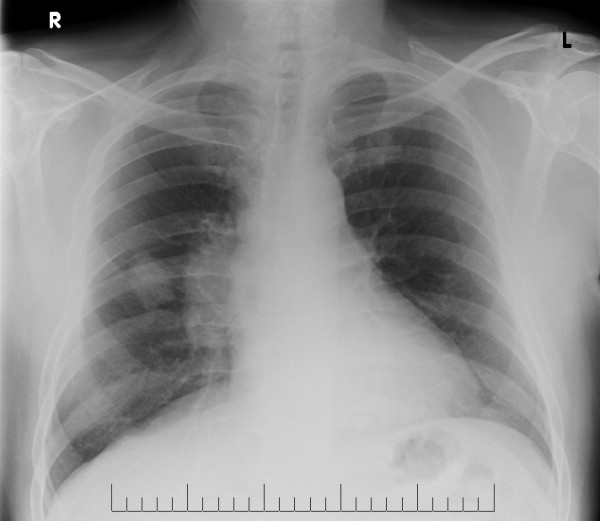
Chest X-ray showing the primary tumour in the right lung.

A chest X-ray showed a right mid zone mass lesion (Figure 2). Routine blood tests including calcium were normal. Alkaline phosphatase was mildly elevated at 126 U/litre (normal upper limit 115 U/litre).

Bronchoscopy showed partial stenosis of his right upper lobe bronchus with some mucosal abnormality. Biopsies and aspirate from this procedure were unfortunately non-diagnostic. Computed tomography (CT) scan confirmed the presence of a right upper lobe mass, along with mediastinal lymphadenopathy and multiple small pulmonary metastases. His bone scan showed increased uptake in the proximal phalanx of his right ring finger and likely metastatic deposits in T9 vertebrae and several ribs.

In order to obtain a histological diagnosis, a biopsy of the finger lesion was performed. This confirmed moderately to poorly differentiated metastatic adenocarcinoma in keeping with lung origin. At a recent oncology review, the patient did not wish to explore chemotherapy treatment options, but went on to have one fraction of palliative radiotherapy to the metastatic deposit in his finger. Unfortunately, he declined further treatment and defaulted from follow-up.

## Discussion

Approximately one-third of patients with lung cancer will have bony metastases at presentation or develop bony lesions during the course of their disease [6]. In the vast majority, these metastases occur in haematopoietically active bones; therefore, metastatic lesions to the bones of the hand are very rare. Hand lesions only account for around 0.1% of all skeletal metastases in malignant disease. Lung cancer is the most frequent primary lesion, followed by breast and kidney [7]. A Medline review of the literature shows reports of metastases to the bones of the hand to be rare, usually occurring where the diagnosis of malignant disease is already known.

## Conclusion

Although this case illustrates an unusual and rare mode of presentation of occult lung malignancy, it highlights the need for a high index of suspicion in patients with persistent bony symptoms and with known risk factors for malignancy.

## Consent

Written informed consent could not be obtained in this case since the patient is untraceable. We believe this case report contains a worthwhile clinical lesson which could not be made as effectively in any other way. We expect that the patient would not object to the publication since every effort has been made so he remains anonymous.

## Competing interests

The authors declare that they have no competing interests.

## Authors' contributions

All authors contributed to the manuscript, MAE and RBG performed the literature review and compiled the original case report. MM was responsible for the care of the patient, initially suggesting the case report and revising the original draft of the manuscript. MAE carried out the final drafting, submissions and revisions process. All authors read and approved the final draft.
